# Efficient Fabrication Process of Ordered Metal Nanodot Arrays for Infrared Plasmonic Sensor

**DOI:** 10.3390/mi10060385

**Published:** 2019-06-08

**Authors:** Masahiko Yoshino, Yusuke Kubota, Yuki Nakagawa, Motoki Terano

**Affiliations:** 1 Mechanical Engineering, School of Engineering, Tokyo Institute of Technology, Tokyo 152-8550, Japan; kubota.y.am@m.titech.ac.jp (Y.K.); nakagawa.y.aw@m.titech.ac.jp (Y.N.); 2 Mechanical Systems Engineering, Faculty of Engineering, Okayama University of Science, Okayama 700-0005, Japan; m_terano@mech.ous.ac.jp

**Keywords:** nanomaterials, plasmonics, nanodot array, thermal dewetting, absorbance spectrum, infrared plasmonic sensor

## Abstract

In this paper, a simple process to fabricate ordered Au nanodot arrays up to 520 nm in diameter that respond to infrared light is developed, and the feasibility of its application to infrared plasmonic sensors is shown. The developed process utilizes thermal dewetting to agglomerate a coated gold film into nanodots. It was difficult to produce large nanodots that responded to infrared light owing to dot separation. In this paper, therefore, the mechanism of dot agglomeration by thermal dewetting is studied via an experiment and theoretical model, and conditions to form single nanodots are clarified. Furthermore, Au nanodot arrays of 100 nm to 520 nm in diameter were fabricated by this process, and their absorption spectra were analyzed. In addition, an analysis of the change in the peak wavelength against the refractive index indicates the possibility of further improvement of the sensitivity of the infrared plasmon sensors.

## 1. Introduction

Metal nano/micro structures smaller than the wavelength of light such as nanodot arrays generate localized surface plasmon resonance by incident light. These structures are expected to be applied to various photo devices, for example, plasmonic chemical sensors using metal nanodots, surface enhanced Raman scattering (SERS) substrates, and devices to enhance luminescence/absorption [1,2,3,4,5,6,7,8,9,10,11]. In particular, their applications to infrared devices have been attracting considerable attention because infrared light interacts with many substances, making it useful for material analysis. Several studies on infrared devices such as infrared band filters [12,13], infrared plasmonic sensors [14,15], heat radiation/absorption control [16,17], and infrared image sensing have been reported.

The electron beam lithography (EBL) process is considered to be suitable to fabricate nano/micro structures [18,19,20,21,22]; however, it requires expensive facilities and stringent control of process conditions, consequently increasing the product’s cost. In addition, hazardous chemicals are necessary for the etching process, rendering this method dangerous in terms of environmental load. Therefore, an efficient and inexpensive method for manufacturing metallic nano/micro structures is required. To address the high product cost problem, many processes have been proposed, including nanoimprinting lithography [23,24], chemical stamp lithography, the porous anode alumina method [25,26], and colloidal lithography [6,27,28,29,30]. However, the capability of size control, precision of dimensions, and productivity are still insufficient for plasmonic sensor applications to be achieved.

The authors developed a simple and efficient manufacturing method for metal nanodot arrays by combining nano plastic-forming and thermal dewetting [31,32,33,34,35]. This method does not utilize EBL and it is not harmful to the environment. They fabricated nanodot arrays of hemisphere metal nanodots ranging from 75 nm to 200 nm in diameter. which exhibited an absorbance peak by localized surface plasmon resonance (LSPR) in the visible light range. It was shown that this peak wavelength could be controlled by adjusting the nano plastic-forming condition, making this method useful for efficient fabrication of plasmonic sensors. In addition, increments in sensitivity and SERS have been achieved by multilayering a metal nanodot array [36,37].

Owing to dot separation in thermal dewetting, these Au nanodot arrays were limited to a diameter of 200 nm or less, and exhibited plasmon resonance in the visible light range. Because of that, they could not be applicable to the infrared sensors that require larger arrays. Despite many studies on thermal dewetting instability, the separation mechanism of the coated metal film and the effects of process conditions on the dot size are not apparent [38,39,40,41]. To stably produce a nanodot array larger than 200 nm and determine optimal process conditions, it is necessary to clarify the principle of dot separation.

In this paper, the authors aim to clarify the principle of thermal dewetting dot separation in their developed process and show a possible application of Au nanodot arrays to infrared plasmon sensors. First, the experimental method and results are explained. Then, theoretical models are proposed to discuss characteristic phenomena of dot agglomeration by thermal dewetting. Through the discussion, the process conditions necessary to fabricate a nanodot array of target size are clarified. Finally, optical properties of the nanodot arrays are analyzed by a spectroscope. Results indicate that fabricated nanodot arrays have an absorption peak in the infrared range, and its peak wavelength depends on the refractive index of the surrounding liquid. There are several advantages of Au nanodot arrays when compared with other metal materials such as the lack of aging variation interference, because Au is hardly oxidized in the atmosphere. As a result, non-oxidation facilities such as vacuum chambers and inert gas chambers are not needed in its manufacturing, consequently reducing the product’s cost. Although the proposed process is also applicable to other noble metals such as Ag and Pt, as previously reported, only Au is used as nanodot material in this paper. The methods and results reported can be also useful for fabrication of nanodot arrays of various metals.

## 2. Fabrication Process for Metal Nanodot Array

**Figure 1:**
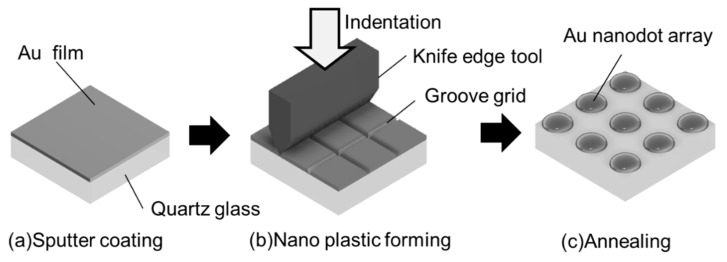
Schematic illustration of proposed fabrication process.

Figure 1 shows the fabrication method for a gold nanodot array. This process consists of three steps: (a) sputter coating of Au film, (b) groove grid patterning on the Au film, and (c) thermal dewetting to form a Au nanodot array [33]. In step (a), a quartz glass plate of 1 mm in thickness, finished by polishing to an optical flat, was used as a substrate. The quartz glass substrate was cleaned ultrasonically in an acetone bath and was then dried in air at ambient temperature. Next, the Au was coated on a quartz glass substrate by using a DC (direct current) sputter coater. The sputtering gas was Ar, and the pressure was 10 Pa. In step (b), a nanogroove grid was machined on Au thin film by repeating step feed and indentation motion of a knife edge too using the nano plastic-forming technique. Motion of the nano plastic-forming device was controlled by a computer, and a groove grid of arbitrary size could be produced. In step (c), the machined quartz glass substrate was annealed in atmosphere using an electric furnace. By this process, the Au thin films sectioned by the groove grid were aggregated into uniform nanodots that were aligned in an orderly fashion at uniform intervals. 

**Figure 2:**
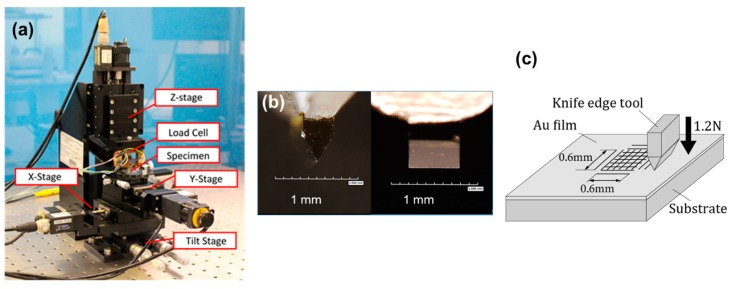
Nano plastic-forming device and knife edge tool made of single crystal diamond. (**a**) whole image of the device; (**b**) optical microscope image of the tool; (**c**) illustration for nanogroove fabrication method.

Figure 2a shows the nano plastic-forming device. It consists of ultra-precision XY stages and a Z stage. Their feed resolutions are 1 nm for the X stage and 10 nm for the Y and Z stages. A specimen is mounted on the XY stage, and a knife edge tool is mounted on Z stage together with a load cell. The knife edge tool is indented onto the specimen by moving the Z stage downward. Figure 2b shows the knife edge tool made of a sharply polished (edge radius smaller than 30 nm) single crystal diamond. The edge’s angle is 60° and the width of the diamond tool is 0.6 mm (more details of the equipment are explained in previous paper [32]). As shown in Figure 2c, a series of parallel nanogrooves are machined on the Au film by indenting the knife edge tool. For this experiment, the indentation load of the knife edge tool is set to 1.2 N. Then, the substrate is rotated 90º horizontally, and another series of parallel grooves are machined on the preformed grooves. Because all motion of the nano plastic forming device are controlled by a computer program, a nanogroove grid of an arbitrary size larger than 100 nm can be machined by this method. Table 1 lists the experimental conditions for examining the characteristics of the aggregation of Au nanodot arrays. 

## 3. Experimental Results and Agglomeration Mechanism by Thermal Dewetting

### 3.1. Agglomeration of a Single Nanodot

**Figure 3:**
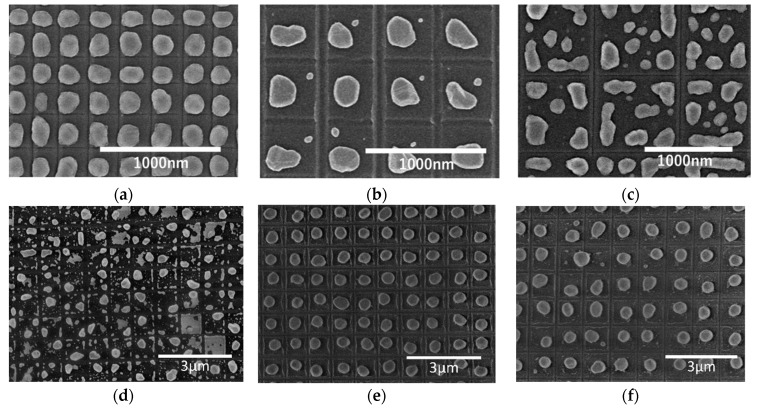
Field-emission scanning electron microscope (FE-SEM) micrographs of gold nanodot array generated by annealing; (**a**) t = 10 nm, P = 250 nm, T = 700 °C; (**b**) t = 10 nm, P = 500 nm, T = 700 °C; (**c**) t = 10 nm, P = 1000 nm, T = 700 °C; (**d**) t = 17 nm, P = 1000 nm, T = 700 °C; (**e**) t = 40 nm, P = 1000 nm, T = 1000 °C; (**f**) t = 40 nm, P = 1200 nm, T = 1000 °C.

Figure 3 shows scanning electron microscopy (SEM) images of the nanodot array. When the groove grid size and the thickness of the gold thin film are appropriate, the Au films in each grid are agglomerated into a single dot forming a uniform dot array with regular intervals (a,e). If the size and thickness are not appropriate, the gold thin film in the groove grid is separated and agglomerated into multiple dots (c,d). In addition, the size of the nanodots changes based on the groove grid size. These dots are well aligned in a lattice pattern, and most of them are formed into an almost hemispherical shape, while some of them are distorted into an ellipsoidal shape. Although the detail of this distortion is not yet fully justifiable, insufficient agglomeration is one of its main causes. It can be reduced by increasing annealing temperature or annealing time. Figure 3d illustrates an example where dot agglomeration is not complete, but the gold film is separated into many pieces, which will be agglomerated into multiple dots. 

**Figure 4:**
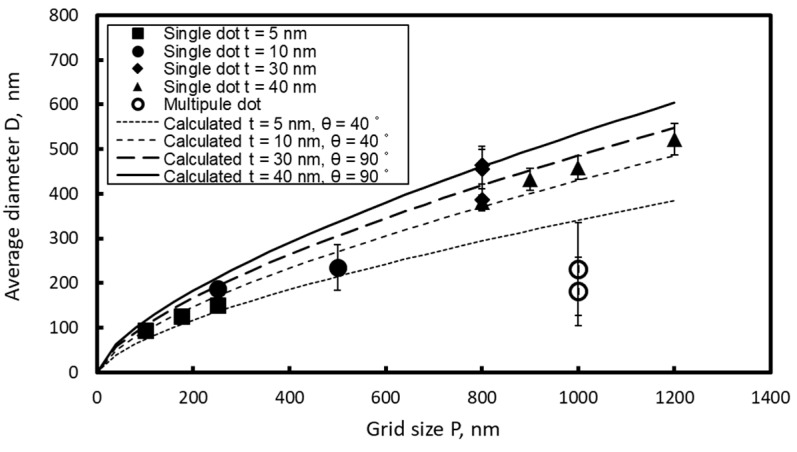
Variation of average nanodot size and its standard deviation against grid size.

Figure 4 shows the variation of the average nanodot diameter with respect to the size of the groove grid. In this figure, the solid symbols indicate the single-dot mode and the open symbols indicate the multiple-dot mode. The vertical bars represent the standard deviation of the dot diameter. Five to 10 specimens were tested on the same condition. In the single-dot mode, the average diameter of the nanodots increases with an increase in the grid size, as shown by the solid symbols. However, in the multiple-dot mode, the average diameter of the divided dots is small, and the standard deviation is large. 

**Figure 5:**
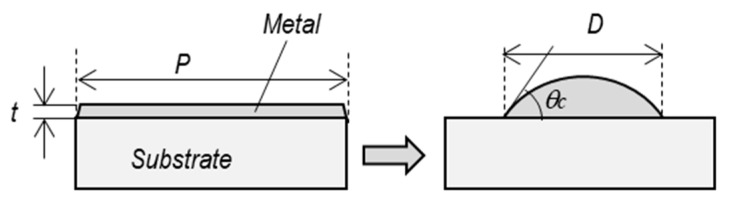
Geometrical change of a nanodot by thermal dewetting.

In the single-nanodot mode, the diameter of the nanodot is calculated based on the volume preservation in the thermal dewetting process. The volume of a hemisphere nanodot is equivalent to the volume of the square film of a groove grid, as shown in the model of Figure 5.

(1) $$\frac{\pi }{24}\frac{D^{3}}{\sin^{3}\theta_{c}}\left(2-3\cos\theta_{c}+\cos^{3}\theta_{c}\right)=P^{2}t$$

The term on the left is the volume of a dot whose contact angle is *θ_c_.* When the agglomeration is completed, *θ_c_* is determined by the balance of the surface energy of the substrate *γ_S_*, the surface energy of the nanodot *γ_M_*, and the interface energy between the metal film and the substrate *γ_I_*. This is expressed by Young’s equation [42]:

(2) $$\gamma_{I}-\gamma_{s}=-\gamma_{M}\cos\theta_{c}$$

From Equation (1), the diameter of the nanodot is obtained as follows:

(3) $$D={\left\{\frac{24P^{2}t}{\pi \left(2-3\cos\theta_{c}+\cos^{3}\theta_{c}\right)}\right\}}^{\frac{1}{3}}\sin\theta \;\;\;\;\;\;\;\left(0{}^{\circ}<\theta_{c}\leq 90{}^{\circ}\right)$$

The curves in Figure 4 represent variations in *D* calculated by Equation (1). In this equation the dot diameter depends on the grid size and the contact angle; dot diameter becomes larger when the contact angle becomes smaller. In Figure 4, the contact angles were adjusted so that the calculated curves agreed with the experimental data: the contact angle *θc* = 40° when *t* = 5 nm and *t* = 10 nm (*T* = 700 °C), and the contact angle *θc* = 90° at *t* = 30 nm (*T* = 1000 °C). It is confirmed that the model explains variation of the nanodot diameter of single mode well. Although this calculation model assumes that all nanodots are agglomerated into a hemispherical form with equal contact angles, the actual nanodots are distorted as seen in Figure 2, and the contact angles varies along the circumference of a dot. Thus, the calculated contact angles do not represent the correct contact angles. However, the contact angles of 40° and 90° determined from the curve fitting can be considered representing the average contact angle of actual nanodots, including some 10° of error. 

Difference between the contact angles of both conditions is attributed to the difference in the annealing temperature. Because thermal dewetting is attributed to atomic diffusion, and the diffusion coefficient depends on temperature, the agglomeration rate increases with annealing temperature. It is also known that the annealing temperature affects the surface energy of the gold and the quartz glass, and also the interface energy between the Au nanodots and quartz glass substrate. Because the contact angle of the Au nanodot depends on balance of these energies, the annealing temperature affects the aggregation process and final geometry of the Au nanodots. However, the contact angle is also affected by atmosphere gas, and influence of annealing temperature is not clear. Thus, we do not discuss the effect of the annealing temperature on the contact angle of the nanodots, but regard the contact angle obtained from the curve fitting in Figure 4 as the practical value of the average contact angle. 

The agglomeration is driven by a reduction in the total free energy of the substrate/metal system. The total free energy of the substrate/metal system before annealing *G*_1_ is calculated as follows:

(4) $$G_{1}=P^{2}\gamma_{M}+P^{2}\gamma_{I}=P^{2}\left(\gamma_{M}+\gamma_{I}\right)$$

The total free energy of a system in the agglomeration process is expressed as the summation of the surface energy of a dot, the interfacial energy, and the surface energy of the exposed substrate.

(5) $$G_{2}=S_{M}\gamma_{M}+S_{I}\gamma_{I}+\left(P^{2}-S_{I}\right)\gamma_{S}$$

where *S_M_* is the area of the dot surface, and *S_I_* is the area of the interface between the dot and the substrate. They are calculated by the following equations:

(6) $$S_{M}=\frac{\pi D^{2}}{2}\frac{1-\cos\theta_{c}}{\sin^{2}\theta_{c}}$$

(7) $$S_{M}=\frac{\pi D^{2}}{4}$$

Using Equations (2), (6), and (7), the total free energy is obtained as follows:

(8) $${G_{2}}^{single}=\frac{\pi D^{2}}{4}\frac{2-\cos\theta_{c}-\cos^{2}\theta_{c}}{1+\cos\theta_{c}}\gamma_{M}+P^{2}\gamma_{S}$$

**Figure 6:**
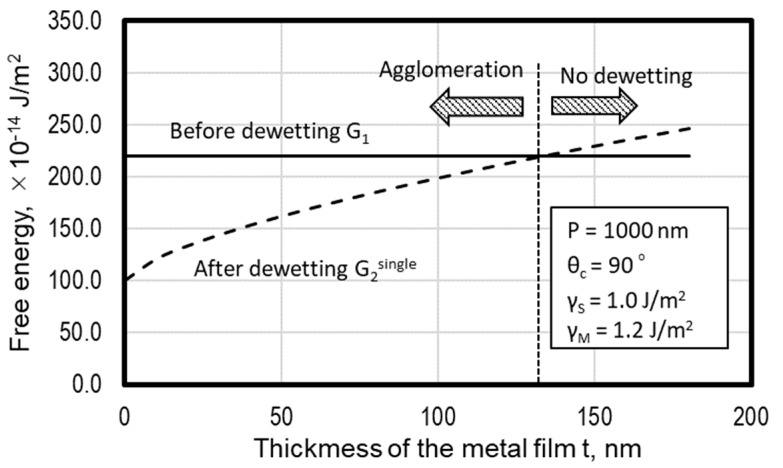
Variation of free energies G_1_ and G_2_^single^ as calculated by Equation (4) and (8).

Figure 6 shows the calculated variation of the free energies *G_1_* and *G_2_^single^*. As for the calculation, the grid size *P* was 1000 nm, and the contact angle *θ_c_* was 90°. The surface energy of Au and SiO_2_ referred to in the literature was adopted as $\gamma_{M}$ and $\gamma_{I}$, respectively. It was shown that the free energy after dewetting *G_2_^single^* was smaller than that before dewetting *G_1_* and nanodots are formed when the thickness of the metal film was small enough. In addition, this equation indicates possibility that agglomeration does not occur when the metal film is too thick. 

### 3.2. Agglomeration Mechanism of Multiple Nanodots

In case of very thin Au film, the film separated and agglomerated into multiple nanodots. This is considered to be the same phenomenon as the normal thermal dewetting process reported by Cheynis et al. [43] because the sectioned square region was relatively broad compared to the film thickness. Danielson [41] explained the agglomerated dot size based on the Rayleigh instability mechanism. However, the Rayleigh instability is a dynamic vibration phenomenon of a stream of falling water [44] and is not suitable for thermal dewetting, which is attributed to the diffusion mechanism. 

**Figure 7:**
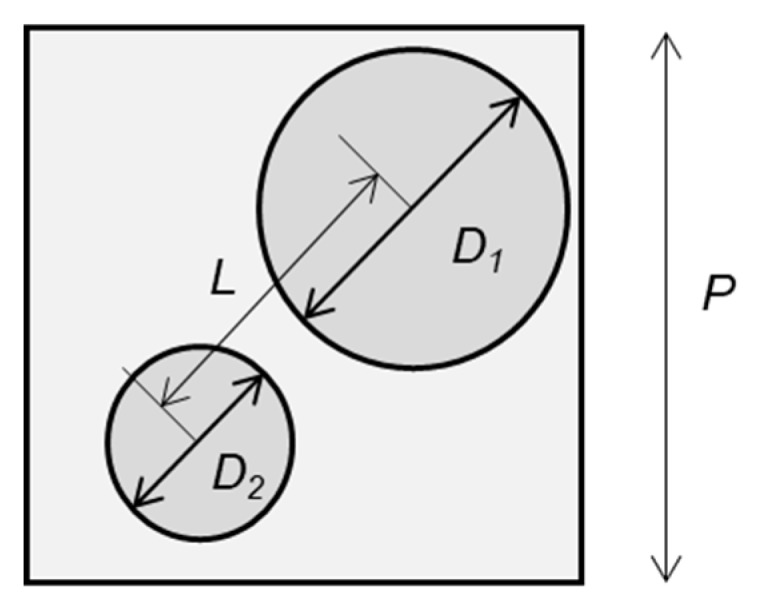
Two nanodots formed in a square grid.

In a typical thermal dewetting process, many microdefects in the metal film [45] are widened by solid diffusion and become voids. These voids grow and connect to adjacent voids owing to thermal dewetting, and create isolated metal islands. Finally, the isolated metal islands agglomerate into hemispherical dots. However, in the experiments in Figure 2, the area of agglomeration is restricted by the groove grids. For dot separation to occur, it is necessary that the dots do not contact each other after aggregation. Here, a case is assumed in which two dots of diameters *D*_1_ and *D*_2_ are formed in a square grid, as shown in Figure 7. 

Because the sum of both volumes is equal to the volume of the coated metal film of thickness *t*,

(9) $$V_{1}+V_{2}=P^{2}t$$

where *V_1_* and *V_2_* are the volumes of these dots. By expressing the ratio of *D*_1_ and *D*_2_ by *k*, i.e., $D_{2}=kD_{1}$. the diameters of these dots are calculated by the following equation:

(10) D2=kD1=k{24sin3θπP2t(2−3cosθ+cos3θ)(1+k3)}130°<θ≤90°

If the distances of the dot centers are shorter than the sum of the radiuses of both dots, then the two dots come in contact and aggregate into one dot owing to the surface tension of the metal. Therefore,

(11) $$\frac{\left(D_{1}+D_{2}\right)}{2}=\frac{\left(D_{1}+kD_{1}\right)}{2}=\frac{D_{1}}{2}\left(1+k\right)>L$$

is the condition of a metal film in a square grid to be agglomerated in a single dot.

If the center of a dot is too close to the boundary, the outer periphery of the dot overlaps the groove, and it cannot be formed into a round dot. Thus, the center of the dot must be apart from the boundary by more than the distance of the radius. Therefore, the region in which a dot can exist is a square area of $L=P-\frac{D_{1}}{2}-\frac{D_{2}}{2}$ on each side. Because the average distance between two points randomly dispersed in a square area is expressed as *μL* ≅ 0.521*L* (see Appendix A), Equation (11) yields

(12) $$\frac{D_{1}}{2}\left(1+k\right)>\mu \left(P-\frac{D_{1}}{2}-\frac{D_{2}}{2}\right)$$

Substituting Equation (10) into Equation (12), we obtain the following inequation:

(13) $$\frac{t}{P}>\left(2-3\cos\theta +cos^{3}\theta \right)\frac{\pi }{3sin^{3}\theta }\frac{\mu^{3}}{{\left(1+\mu \right)}^{3}}\frac{\left(1+k^{3}\right)}{{\left(1+k\right)}^{3}}$$

Hereinafter, *t/P* is called the critical film thickness ratio. It is considered that a single dot is generated when *t/P* is larger than the critical film thickness ratio, and multiple dots are generated when *t/P* is smaller than the critical film thickness ratio.

**Figure 8:**
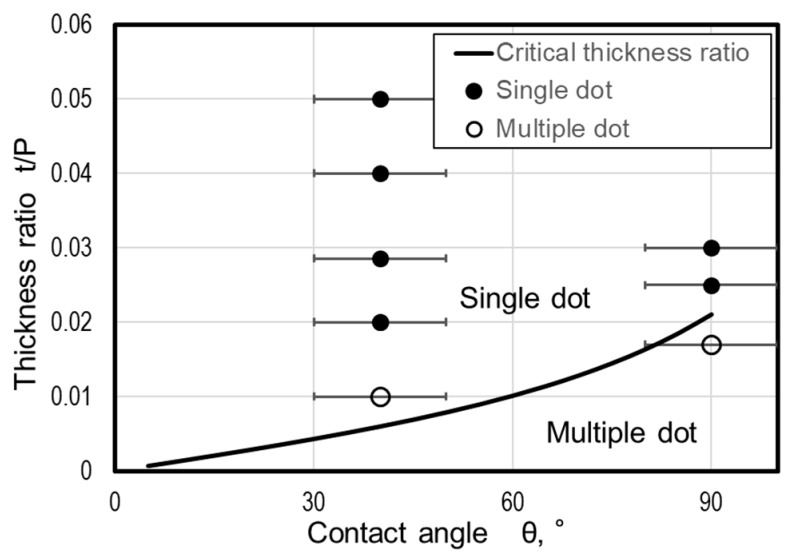
Comparison between theoretical value of critical film thickness ratio and experimental value of dot formation.

Figure 8 shows the variation of critical film thickness ratio calculated by Equation (13) (curve) and the experimental data in which single (solid symbols) and multiple (open symbols) dots were formed, against the contact angle. For reference, a contact error of range ±10° is also displayed. The solid line lies near the boundary between the white circles and the black circles; therefore, the model correctly explains the condition of the single-dot mode and multiple-dot mode the error associated with the prediction is considered due to the distortion of nanodots. Even when there are more than three dots, the critical film thickness is determined by a similar condition that dot diameter must be larger than the average of minimum distance between dots. Although details of calculations are not shown here, the obtained result proves that the critical film thickness ratio decreases as the number of dots in the lattice increases.

Figure 8 also indicates that the critical film thickness ratio increases with the contact angle. A single nanodot correctly aligned as the groove grid can easily be obtained in case of a substrate and metal with small contact angle. Although it is difficult to control the contact angle because it depends on the combination of materials and the annealing conditions, the dot size can be controlled by adjusting the film thickness ratio to a range larger than critical film thickness ratio.

The total free energy of the dot/substrate system of the multiple-dot mode is also calculated by Equation (5), where *S_M_* and *S_I_* are calculated by the following equations:

(14) $$S_{M}=\frac{\pi \left({D_{1}}^{2}+{D_{2}}^{2}+\cdots +{D_{N}}^{2}\right)}{2}\frac{1-\cos\theta_{c}}{\sin^{2}\theta_{c}}=\frac{\pi \sum_{i=1}^{N}{D_{i}}^{2}}{2}\frac{1-\cos\theta_{c}}{\sin^{2}\theta_{c}}$$

(15) $$S_{I}=\frac{\pi \left({D_{1}}^{2}+{D_{2}}^{2}+\cdots +{D_{N}}^{2}\right)}{4}=\frac{\pi \sum_{i=1}^{N}{D_{i}}^{2}}{4}$$

where *N* is the number of dots in a square grid area. The total free energy of the system is obtained as follows:

(16) $${G_{2}}^{multiple}=\frac{\pi \sum_{i=1}^{N}{D_{i}}^{2}}{4}\frac{2-\cos\theta_{c}-\cos^{2}\theta_{c}}{1+\cos\theta_{c}}\gamma_{M}+P^{2}\gamma_{S}$$

**Figure 9:**
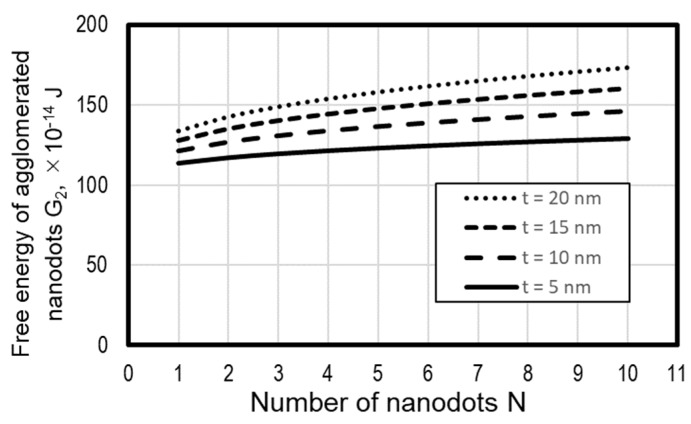
Variation of free energy of separated dots against the number of dots in a square grid.

Figure 9 shows the calculated variations of the free energy against the number of dots, where all of the dot sizes are assumed to be equal and the material data of Au and quartz glass are adopted. It is reasonable that the free energy increases with the number of nanodots. It is also found that the free energy increases with an increase in the metal film thickness. This is because an increase in film thickness results in an increase in the dot diameter and thus an increase in S_M_ of Equation (11). When the film becomes thicker, the increase in free energy against the increase in the dot number becomes larger and, therefore, a thick film is easier to agglomerate to a large dot. 

Because a lower free energy increases the possibility of the phenomenon’s occurrence, nanodots are agglomerated into a single dot only if a reduction in the free energy governs the dot agglomeration. However, the aggregation of the dots results from the solid diffusion of atoms in the metal film, and it is difficult for an aggregation pattern that needs a long migration of atoms to occur. In addition, it is known that a large number of microdefects in the metal film grow into many holes, and they separate the metal film by thermal dewetting. Therefore, it is understood that the coated metal film is agglomerated into multiple dots if there is enough space between dots, but in the case of the sectioned metal film of our process, the number of dots is restricted by the condition of the critical film thickness ratio in Equation (13).

## 4. Optical Properties of Au Nanodot Arrays

**Figure 10:**
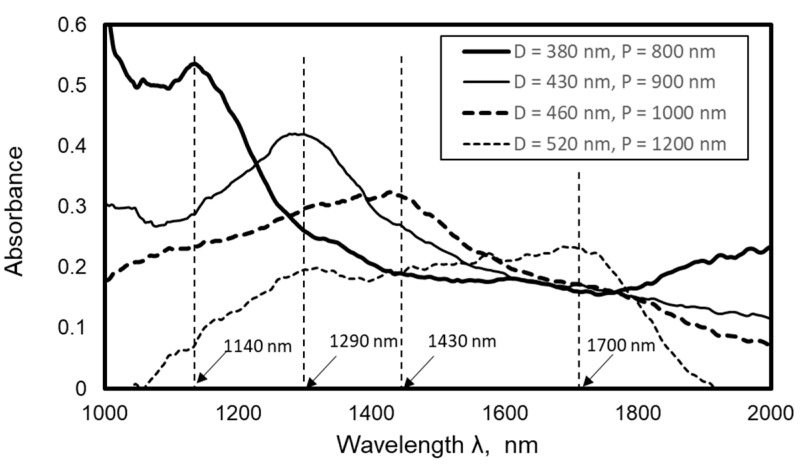
Influence of average dot diameter on absorbance spectrum of nanodot arrays.

The absorbance spectra of the fabricated Au nanodot arrays were analyzed by using a fiber-type infrared (IR) spectrometer whose area of analysis was almost 0.5 mm in diameter. In addition, three kinds of liquid, i.e., water, acetone, and kerosene, were dropped on nanodot arrays, and the absorbance spectra were analyzed to evaluate the influence of the refractive index. Figure 10 shows the absorbance spectrum of an Au nanodot array measured by a fiber-type spectrophotometer. In the figure, a clear peak is observed in the near-infrared region, which is considered to be owing to localized plasmon resonance. It is found that the peak wavelength increases with increase of the dot diameter. 

**Figure 11:**
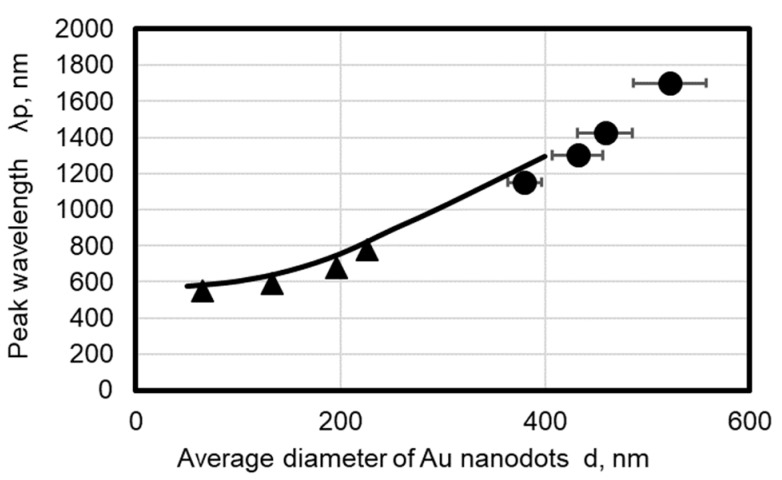
Change in peak wavelength against average dot diameter. ●: data obtained from Figure 10, ▲: data referred from a previous report [32], curve: calculated date.

Figure 11 shows the change in the peak wavelength with respect to the average diameter. Solid circles represent data of Figure 10 and solid triangles represent data obtained from a previous report [35]. It is found that the peak wavelength increases almost linearly with an increase in the average dot diameter *D*.

The solid line in Figure 11 is the modeled variation of peak wavelength of the absorbance spectrum based on the Kuwata’s method [46]. In this model, the extinction cross section of an ellipsoidal nanodot, whose radius is denoted by a, b, and c, is calculated by

(17) $$\sigma_{ext}=k\cdot Im\left(\alpha \right)$$

where k is the wave number of the incident light calculated by

(18) k=2π(εm)12λ

$\varepsilon_{m}$ is the dielectric constant around the nanodot, which is assumed as the average of the relative permittivity of quartz glass substrate and air [47]. λ is the wavelength of the incident light. α is the polarizability expressed by the following equation [46]:

(19) $$\alpha =\frac{V}{\left(L+\frac{\varepsilon_{m}}{\varepsilon -\varepsilon_{m}}\right)+A\left(L\right)\varepsilon_{m}x^{2}-i\frac{4\pi^{2}{\varepsilon_{m}}^{3/2}V}{\lambda^{3}}+B\left(L\right){\varepsilon_{m}}^{2}x^{4}}$$

where ε is the complex dielectric constant of the nanodot metal, i.e., Au [48], *x* is the size parameter of an ellipsoidal nanodot (x=πa/λ), and *V* is the volume of the ellipsoidal nanodot. In the case of an oblate spheroid (a = b = 2c, where c is the radius to incident light direction), the geometrical factor *L* is calculated by the following equation [49]:

(20) L=12e2(1−e2e2)12{π2−tan−1(e(1−e2e2)12)}−12(1−e2e2)

where e is the eccentricity.

(21) $$e^{2}=1-\frac{c^{2}}{a^{2}}$$

*A(L)* and *B(L)* in equation (16) are calculated by the following equations

(22) $$A\left(L\right)=-0.4865L-1.046L^{2}+0.8148L^{3}$$

(23) $$B\left(L\right)=0.01909L+0.1999L^{2}+0.6077L^{3}$$

Using these equations, absorbance spectra of an oblate spheroid of various sizes is simulated and compared with the measured peak wavelength of the absorbance spectra. The simulation data in Figure 11 agrees well with the experimental data; thus, the absorbance peak found in Figure 10 can be attributed to the localized surface plasmon resonance. The residual difference between the simulation data and experimental data is due to the difference of dot shape because the actual Au dot fabricated by the experiment was not an oblate spheroid but a distorted hemisphere. 

Another important conclusion from the theoretical model is that the absorbance peak wavelength due to the localized surface plasmon resonance also depends on the shape of the dot, i.e., the ratio of a, b, and c. As a result, the width of the absorbance spectrum peak spreads and peak wavelenght becomes unclear. Thus, it is important to reduce distortion of shape and size dispersion of nanodots to improve sharpness of the absorbance spectrum peak and accuracy of peak wavelength. If a sharp absorbance peak can be realized by this process, it can be used as an infrared filter. In order to detect a signal of a target wavelength with high sensitivity in material analysis by infrared (e.g., Fourier transform infrared (FT-IR)), it is necessary to reduce signal noise. An infrared filter of the Au nanodot array with required absorbance peak wavelength can be made by controlling the Au dot size by adjusting the nano groove grid size and the Au film thickness. Using this Au nanodot filter, noise can be appropriately reduced and the target signal can be successfully measured. Furthermore, the Au nanodot arrays can be utilized as an absorber of heat radiation energy.

**Figure 12:**
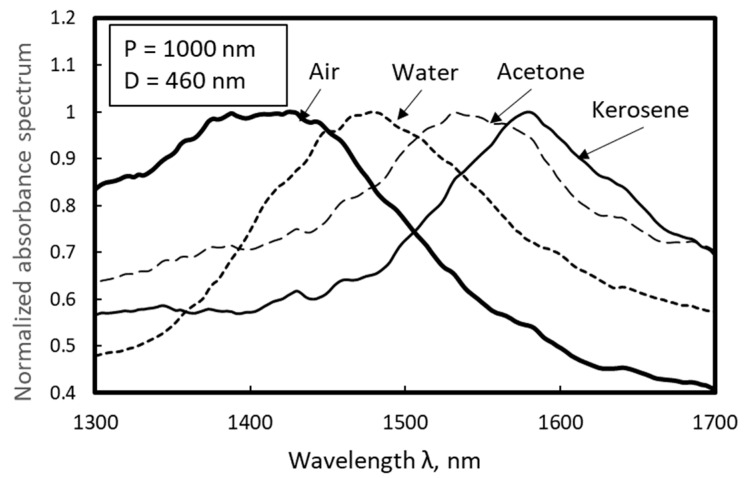
Effects of refractive index on absorbance spectra of nanodot array of 460 nm in diameter.

Figure 12 shows the effects of the refractive index on the normalized absorbance spectra of a nanodot array of 459 nm in diameter, where water (refractive index n = 1.33), acetone (n = 1.36), and kerosene (n = 1.45) were dropped; data without liquid specimen, i.e., air (n = 1) are also shown. It is apparent that the peak of the absorbance spectrum shifted to the red side when the refractive index was modified by the dropped liquid. This characteristic is utilized in plasmonic sensors; the refractive index of a liquid specimen can be evaluated by a shift in the peak wavelength, which can be measured by the simple setup of a fiber-type spectroscope. This technology can be also applied to sensing of biomolecules such as protein or viruses.

**Figure 13:**
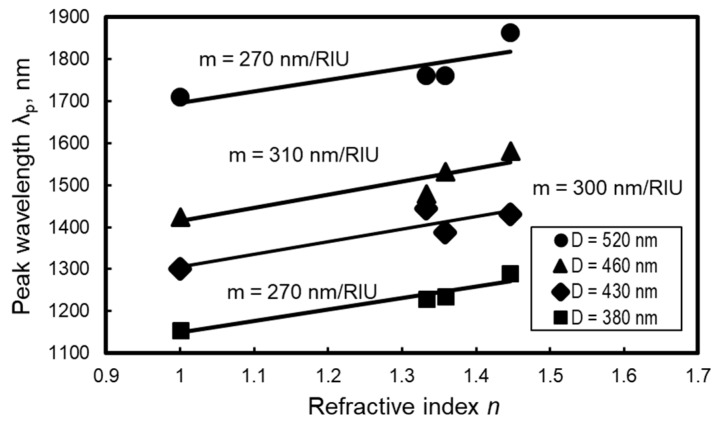
Change in peak wavelength against refractive index.

Figure 13 shows the variation in the peak wavelength based on the refractive index of the dropped liquid. The gradient of the approximated line (m-value) is also denoted in the graph. The m-value indicates the change in the peak wavelength of the absorbance spectrum (nm) against the change in the refractive index of the dropped liquid (RIU, refractive index unit). From Figure 13, it is found that the peak wavelength linearly increases with the dropped liquid’s refractive index. It is also found that the m-value depends on the average dot diameter *D*. 

**Figure 14:**
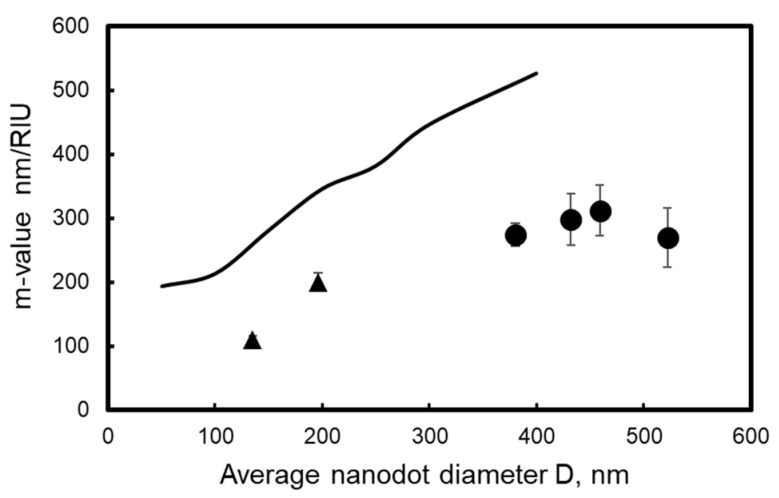
Change in sensitivity against average dot diameter. ●: data obtained from Figure 13, ▲: data referred from a previous report [35], curve: calculated date.

Figure 14 plots the m-value against the average dot diameter. The solid circles are data of m-values obtained from Figure 13, and the solid triangles are data of m-values in the visible light range referred from a previous report [34]. The figure shows that m-values obtained from Figure 13 are higher than those of the previous report, indicating that larger dots exhibit a higher m-value and a higher m-value can be obtained in the infrared range than in the visible light range. The line in Figure 14 represents the peak wavelength’s variation in simulated absorbance spectra using Kuwata’s method. The simulation results present a peak wavelength increase with dot diameter. This behavior is similar to the experimental data; however, the calculated m-value is almost twice as large. This is attributed to the difference in shape and the difference in the layout of the dot between the actual nanodots and the calculated model because actual nanodots are distorted hemispheres on the quartz glass. Nevertheless, the fundamental LSPR properties can still be explained by Kuwata’s method. It is apparent that larger nanodots generate a higher m-value that is more likely to be obtained in the infrared range than in the visible light range. As the proposed fabrication process is able to control the size and geometry of the nanodot array, the sensitivity of plasmon sensors can be improved by developing suitable dot geometry for analysis by infrared light.

The nanodot array is expected to be applied in a chemical sensor that can analyze the component of a liquid specimen by measuring the refractive index based on the change in the absorption spectrum peak wavelength. To develop a sensitive chemical sensor, a higher m-value is preferable; therefore, a large nanodot array with a spectral peak in the infrared region is advantageous. Furthermore, a sharp absorption spectrum peak is preferable, and it is required to fabricate uniform nanodot arrays with small dispersion in dot diameter and little distortion in shape. For this purpose, further understanding of the thermal dewetting mechanism and development of control technology of the agglomeration process is necessary.

## 5. Conclusion

This study examined a simple and low-cost process for the fabrication of uniform metal nanodot arrays by combining nano plastic forming and thermal dewetting by annealing. The process conditions were clarified to produce relatively large nanodot arrays with a diameter up to 520 nm; these arrays could be useful for infrared light analysis. 

The original process proposed faced a problem because the metal film had been separated into multiple dots during the thermal dewetting as the size of the nanogroove grid became large. In this study, therefore, a geometrical model to explain the mechanism of dot aggregation and separation by thermal dewetting was developed, and conditions for aggregation into single dots on each grid were clarified. The model was validated by comparing the results with the experimental data, enabling the fabrication of Au nanodot arrays of various dot sizes (from 100 nm to 520 nm). Although problems still remain with uniformity, distortion, and dispersion in size and geometry of nanodots, the feasibility of the process was confirmed.

Furthermore, it was shown that an Au nanodot array produced by this process exhibits an absorbance spectrum in the visible light to infrared range peak owing to the localized plasmon resonance. Because the peak wavelength of the absorbance spectrum depends on the average dot diameter, the proposed technology is effective to develop optical devices with various absorbance peaks. In addition, it is important to reduce the distortion of dots to obtain a unique absorbance peak as the distortion of the dot influences the peak wavelength. 

Moreover, it was shown that the peak wavelength also depends on the liquid’s refractive index dropped on the nanodots. A shift in the peak wavelength against change in the refractive index, i.e., m-value, was found to increase with average dot diameter. Because a high m-value is preferable for sensor sensitivity improvement, a large nanodot array with a spectral peak in the infrared region is advantageous for developing a highly sensitive sensor. The proposed fabrication process is, therefore, expected to improve the sensitivity of plasmon sensors by improving the dot geometry suitable for analysis by infrared light.

## Figures and Tables

**Table 1:** Experimental conditions of first-layer nanodot array.

Thickness of Gold Film t, nm	Grid Size P, nm	Annealing Temperature T, °C	Annealing Time, min
5	50, 75, 100, 175, 250	700	10
10	250, 500, 1000	700	10
30	800, 900	1000	10
40	1000, 1200	1000	10

## Appendix A

The average distance between two points randomly dispersed in a unit square is calculated by the following equation:

$$\mu =\int_{0}^{1}\int_{0}^{1}\int_{0}^{1}\int_{0}^{1}\left\{\sqrt{{\left(x_{1}-x_{2}\right)}^{2}+{\left(y_{1}-y_{2}\right)}^{2}}\right\}dx_{1}dx_{2}dy_{1}dy_{2}=\frac{1}{15}\left\{2+\sqrt{2}+5\mathrm{arcsinh}\left(1\right)\right\}≅0.521$$

In the case that more than three points exist in a unit square, the average of the minimum points’ distance is obtained by a numerical simulation in which a determined number of points were randomly distributed in a unit square by a random function, and then the minimum distance between them was calculated. By repeating this calculation more than 1,000,000 times, the average minimum distance was obtained. Table A1 shows the average of the minimum distances and standard deviations against the number of points used.

**Table A1:** Average of the minimum distances and standard deviations against the number of points.

Number of Points	Average of Minimum Distance	Standard Deviation
2	0.52106	0.24783
3	0.30556	0.16004
4	0.21179	0.10976
5	0.16233	0.08362
6	0.13189	0.06816
7	0.11103	0.05737
8	0.09583	0.04959
9	0.08436	0.04367
10	0.07538	0.03904
